# Supplementary material for: RNA velocity unraveled
Source: PLoS Comput Biol. 2022 Sep 12;18(9):e1010492. doi: 10.1371/journal.pcbi.1010492 (PMC9499228; doi:10.1371/journal.pcbi.1010492)
Supplement: S1 Text — (PDF) [file pcbi.1010492.s001.pdf]

Supplementary Derivations

A Derivation of the RNA velocity formula

In the current section, we derive the definition of RNA velocity as an instantaneous derivative. The continuous version in Equation 6 can be treated as an axiomatic ODE. Alternatively, it can be formalized as a somewhat singular application of Equation 1 for a Dirac delta measure (a single observed cell with a concentration of u unspliced and s spliced RNA).

The discrete case requires slightly more statistical machinery to arrive at the same answer. The CME takes the following form:

$$\begin{aligned}\frac{dP(x_u, x_s, t)}{dt} &= \text{[generic transcription master equation]} \\ &+ \beta \left[(x_u + 1)P(x_u + 1, x_s - 1, t) - x_u P(x_u, x_s, t)\right] \\ &+ \gamma \left[(x_s + 1)P(x_u, x_s + 1, t) - x_s P(x_u, x_s, t)\right].\end{aligned} \tag{1}$$

As suggested by the notation, the form of the process upstream of $\mathcal{U}$ is immaterial. By the linearity of expectation:

$$\sum_{x_u, x_s=0}^{\infty} \left[x_s \frac{dP(x_u, x_s, t)}{dt}\right] = \frac{d\left[\sum_{x_u, x_s=0}^{\infty} x_s P(x_u, x_s, t)\right]}{dt} = \frac{d\mu_s(t)}{dt}, \tag{2}$$

which is the derivative of the expectation. Therefore, repeating the summation on the right-hand side, we find:

$$\begin{aligned}\sum_{x_u, x_s=0}^{\infty} x_s(x_s + 1)P(x_u, x_s + 1, t) &= \sum_{x_u=0}^{\infty} \sum_{x_s=1}^{\infty} x_s(x_s - 1)P(x_u, x_s, t) \\ &= \sum_{x_u, x_s=0}^{\infty} x_s(x_s - 1)P(x_u, x_s, t)\end{aligned} \tag{3}$$

because the $x_s = 0$ term is identically zero. Similarly,

$$\begin{aligned}\sum_{x_u, x_s=0}^{\infty} x_s(x_u + 1)P(x_u + 1, x_s - 1, t) &= \sum_{x_u=1}^{\infty} \sum_{x_s=-1}^{\infty} x_u(x_s + 1)P(x_u, x_s, t) \\ &= \sum_{x_u, x_s=0}^{\infty} x_u(x_s + 1)P(x_u, x_s, t),\end{aligned} \tag{4}$$

since $P(x_u, x_s, t) = 0$ whenever x_u or x_s are less than zero. Therefore, the RHS has the following terms in the summation over $x_u, x_s \in \mathbb{N}_0$:

$$\begin{aligned}&P(x_u, x_s, t) \left[\beta x_u(x_s + 1) - \beta x_u x_s + \gamma x_s(x_s - 1) - \gamma x_s^2\right] \\ &= P(x_u, x_s, t) \left[\beta x_u - \gamma x_s\right],\end{aligned} \tag{5}$$

i.e., summing over the microstates with weights $P(x_u, x_s, t)$, we find the RHS has the following term:

$$\beta\mu_u(t) - \gamma\mu_s(t). \tag{6}$$

The specific form of the generic transcription master equation is immaterial, as long as spliced molecules are not directly transcribed. For example, if the transcription is constitutive with transcription rate α, we find

this component and its sum takes the following form:

$$\alpha(t)\left[P(x_u-1,x_s,t)-P(x_u,x_s,t)\right]$$
$$\sum_{x_u,x_s=0}^{\infty} x_s P(x_u-1,x_s,t) = \sum_{x_u,x_s=0}^{\infty} x_s P(x_u,x_s,t), \tag{7}$$

i.e., the influx and efflux terms cancel. Therefore, we find that the usual relationship holds:

$$\frac{d\mu_s(t)}{dt} = \beta\mu_u(t) - \gamma\mu_s(t). \tag{8}$$

Next, we instantiate a degenerate measure with PMF $\delta_{x_u,u}\delta_{x_s,s}$, representing a single cell. The entire probability mass of this measure lies at u, s, thus $\mu_u(t) = u$ and $\mu_s(t) = s$. By plugging these values into Equation 8, we obtain the instantaneous velocity. Per Equation 2, this velocity is the derivative of the expectation of the spliced counts. This proves Equation 6.

B Model-agnostic count correction is impossible

In the body of this report, we discuss problems with the heavy-handed data treatment in the velocity workflows. In "Count processing" under "Logic and methodology," we point out that it is inappropriate to fit an ODE model to normalized, pooled data: doing so amounts to presupposing a result that should be proven using careful theoretical analysis. In the corresponding subsection under "Prospects and solutions," we look for at least informal signs of this result in simulated data, and find nothing of the sort: imputation makes plausible-looking trajectories that nevertheless have very little in common with the ground truth. In the current supplement, we point out a much more general, yet elementary result that implies that count "correction" through imputation can be arbitrarily wrong.

Suppose we have a data point $\mathcal{D}_{ij}$ and the corresponding true mRNA abundance n_{ij} for a particular molecular species, cell i, and gene j. Sequencing is not perfect: the data point $\mathcal{D}_{ij}$ is generated from n_{ij} according to a non-deterministic schema, with an unknown probability law $P(\mathcal{D}_{ij}|n_{ij})$.

Two problems emerge. First, a point estimate of n_{ij} based on observed $\mathcal{D}_{ij}$ is necessarily incomplete: the sequencing process induces an entire distribution of possible n_{ij}. This conditional distribution is given by Bayes' formula:

$$P(n_{ij}|\mathcal{D}_{ij}) = \frac{P(\mathcal{D}_{ij}|n_{ij})P(n_{ij})}{P(\mathcal{D}_{ij})}. \tag{9}$$

Assigning a single value is questionable, and downplays the effects of uncertainty. This remains a problem even if a theoretically optimal choice is taken, such as the point estimate $\text{argmax}_{n_{ij}} P(n_{ij}|\mathcal{D}_{ij})$.

Second, the conditional distribution depends on $P(n_{ij})$, the actual ground truth distribution. This distribution is unknown and needs to be identified and fit based on the data. Therefore, an imputation procedure that assigns a point estimate without considering the underlying distribution is *a priori* distortive.

In other words, this Bayesian argument illustrates that meaningful count correction is impossible without identifying and fitting the data-generating model, which encodes biological effects in $P(n_{ij})$ and technical effects in $P(n_{ij}|\mathcal{D}_{ij})$. Count correction is strictly less powerful than parameter estimation for the biological and technical models, because count correction requires those parameters, whereas knowledge of the parameters immediately implies the entire distribution of the biological and observed variables.

C Challenges of graph-based methods

Throughout this report, we have discussed k-nearest neighbor graphs in the context of RNA velocity and embeddings, incorporating some comparisons to simple linear projections but omitting explicit discussion

of the k-NN procedure and its assumptions. As k-NN is ubiquitous in scRNA-seq, and its applications are manifold, a full analysis is not feasible. In this supplement, we discuss a set of purely theoretical pitfalls which may limit the utility or interpretability of such graphs, leaving validation on simulated data to future work.

A k-NN graph purports to reflect relationships between cells based on similarity between their transcriptomic "states." These states are typically spliced copy numbers that have undergone several steps of count processing, including size-normalization, log-transformation, filtering, and projection onto the top few principal components. The determination of neighbors in this space represents an uncomfortable compromise: if there are too few dimensions, the projection may be unrepresentative of the underlying data matrix; if there are too many, it may be skewed by the "curse of dimensionality." Such distortions are evident in Fig 6.

More subtly, it is unclear that observed transcriptomic similarity between barcodes should imply similarity between cells. *In silico* UMI counts have been filtered through the random process of sequencing; the true underlying transcriptomic state is unknown, and cannot be precisely reconstructed (as outlined above, under Section B in S1 Text). We anticipate that certain narrow problems, such as cell type identification, may be insensitive to this source of error. For example, it is possible that simulated benchmarks can provide empirical results in the vein of "assuming transcriptional dynamics are bursty, cell types are distinguished by at least ten marker genes, these marker genes have an expression differential of at least one order of magnitude, and each cell type comprises 10–20% of the entire dataset, a community detection-based algorithm has a 80% classification accuracy according to a particular metric, which falls to 75% if a particular noise model is imposed." However, at this time, constructing undirected k-NN graphs based on imperfectly observed data appears to have limited theoretical or empirical justification.

The construction of directed k-NN graphs, as proposed in the original publication [1], is considerably more problematic. A directed graph implies a causal relationship between observed cell states; in the implementations of RNA velocity, this causal relationship is Markovian, with a transition rate governed by the alignment between velocities and neighbor directions. We can analyze potential issues in this reasoning by gradually increasing the complexity of the system under analysis.

First, suppose that the RNA dynamics and the chemistry of sequencing are non-random, whereas the cell observations are independent draws from an underlying (deterministic and perfectly known) phase plot. Intuitively, building a directed graph raises questions: one cell is not the "descendant" of another, as both cells were captured and sequenced simultaneously. In this model formulation, the observed cells do not have causal relationships at all. We can build a graph that connects each cell to the neighbors of its position at Δt; at this point, the function used to define the graph is deliberately left generic. Even in this ideal-case scenario, this graph is highly dependent on the value of Δt and strictly less informative than the dynamical system parameters, as its construction requires those parameters.

Next, suppose that RNA dynamics are still deterministic, but sequencing injects noise into the observations. In the ideal case, where the dynamical system and sequencing noise parameters are perfectly known, extrapolating from the current state: the true RNA abundance is unknown. At best, it is possible to instantiate a set of trajectories conditional on all possible latent states. As before, these trajectories depend on the time horizon Δt. Aggregating the trajectories by assigning a weight to a single graph edge is strictly less informative than reporting the trajectories; that, in turn, is less informative than reporting the system parameters. The question of the amount of error incurred by this approach is coarsely equivalent to the question of a hidden Markov model's (HMM's) approximability by a Markov model. Omitting uncertainty due to technical noise is equivalent to assuming a HMM can be effectively described without latent states. This assumption may be approximately valid (e.g., in the limit of perfect sequencing), or grossly incorrect, with no apparent *a priori* way of constraining error.

Suppose now that RNA dynamics of N molecular species are stochastic on the state space $\Omega := \mathbb{N}_0^N$; the observation process is immaterial. If we sample M cells, the microstates corresponding to these data make up a subset Ω_D, such that $|\Omega_D| \leq M$. If the data are generated by a biological Markov process on Ω, a

process truncated to Ω_D will either not be Markov, or fail to recapitulate features of the biological process. This generally holds when $|\Omega_D| < |\Omega|$. The extent of incurred error may vary from minimal to egregious, and cannot be constrained without further knowledge of the system.

This argument has fairly severe consequences and foundations. Defining a directed graph on Ω_D is superfluous: Ω_D is itself constructed out of samples from the traversal of a directed graph isomorphic to the biological continuous-time Markov chain (CTMC). This CTMC is isomorphic to the CME. If states $i, j \subseteq \Omega_D \subseteq \Omega$ have the nonzero rate k_{ij} for the $i \to j$ transition, it is possible to construct an approximating CTMC on Ω_D merely by setting the rate of the corresponding transition to k_{ij}. However, if either one of those states is not in Ω_D, some dynamics are lost. The question of the amount of error incurred by truncation is roughly equivalent to the question of a given infinite CTMC's approximability on a finite subdomain. For broad classes of CME models, finite approximations can do arbitrarily well: truncation to a finite subdomain Ω_D incurs an error governed by the amount of probability flux to and from states outside this domain, and converges (in distribution) to the true CTMC as $|\Omega_D| \to \infty$. The Finite State Projection (FSP) algorithm [2] exploits this approach to evaluate CME solutions. However, the FSP is adaptive, and expands Ω_D on a grid until a desired precision is achieved, rather than using a relatively small set of points which do not necessarily have transitions in the underlying CTMC.

The lack of these direct transitions between observed states in Ω_D implies that a CTMC on Ω cannot be projected down to Ω_D. This principle can be demonstrated using a striking trivial case. Consider a three-state Markov chain:

$$0 \xrightarrow{k_{01}} 1 \xrightarrow{k_{12}} 2. \tag{10}$$

The full state space is $\Omega = \{0, 1, 2\}$. Consider a case where states 0 and 2 are observed in multiple independent chains at some time t, i.e., $\Omega_D = \{0, 2\}$. We wish to define a neighborhood relationship between observations in state 0 and those in state 2, and summarize it as a CTMC on Ω_D. The following results emerge immediately.

Even with perfect knowledge of the original CTMC, a truncated CTMC will not fully recapitulate its dynamics. The residence time in state 0 is exponentially distributed:

$$f(\tau) = k_{01} e^{-\tau k_{01}}. \tag{11}$$

The true transition from 0 to 2 has a hitting time distribution is described by a hypoexponential law:

$$f(\tau) = \frac{k_{01} k_{12}}{k_{01} - k_{12}} \left[e^{-\tau k_{12}} - e^{-\tau k_{01}} \right]. \tag{12}$$

The transition from 0 to 2 on Ω_D – equivalent to the residence time in state 0 – is constrained to be exponentially distributed:

$$f(\tau) = k_{02} e^{-\tau k_{02}}. \tag{13}$$

As the exponential distribution has one parameter, it can match the true residence time distribution, or a single moment of the hitting time distribution, but not both. If we match the residence time distribution, the approximation becomes arbitrarily good as $k_{12} \to \infty$ and arbitrarily poor as $k_{12} \to 0$. The latter case makes not observing the long-lived state 1 somewhat improbable. However, as system dimensionality grows – e.g., if multiple independent CTMCs are started – the intermediate state will be unobserved in at least one of those chains almost surely.

Even with perfect knowledge of the original CTMC, a generic stochastic process will not fully recapitulate its dynamics. We can define a non-Markovian process on Ω_D that will have a hitting time distribution given by Equation 12. However, its residence time will fail to be distributed per Equation 11. By constraining the process to traverse only observed states, the contributions from unobserved intermediate states are omitted, with error that cannot be easily bounded.

This inability to "compress" CTMCs into a smaller domain can also be treated in a more generic way. To define transitions between states, we must assign a single number – the rate – to the transition. Intuitively, we expect that the rates of CTMCs on Ω_D should reflect the relative probabilities of transitions between states in the original CTMC on Ω. Thus, given three states i, j, and l, we would like to impose the following criterion:

$$P(j,t;i,0) > P(l,t;i,0) \; \forall \; t \in \mathbb{R}_+ \implies k_{ij} > k_{il}. \tag{14}$$

where P refers to full CTMC's probability of being in a state j or l at time t, conditional on being in state i at time 0, and k are rates in the "compressed" CTMC. This criterion appears to be the only "natural" one, and it induces a partially ordered set, which is insufficient to even order the transition rates in the CTMC on Ω_D.

In conclusion, graph-based methods are problematic for representing relationships between cells. They can represent certain aspects of dynamics, but inevitably contradict the underlying graph that governs the biophysical CTMC. It is possible to make them agree, albeit only by considerably expanding the graph beyond observed states, recapitulating the CME. In other words, the only cell-cell graph which can quantitatively summarize Markovian biological processes is the graph underlying the CME, with an infinite number of states, remaining forever out of reach and recalling Borges's and Carroll's dichotomy of the map and the territory [3, 4]: "...we now use the country itself, as its own map, and I assure you it does nearly as well."

References

[1] La Manno G, Soldatov R, Zeisel A, Braun E, Hochgerner H, Petukhov V, et al. RNA velocity of single cells. Nature. 2018;560(7719):494–498. doi:10.1038/s41586-018-0414-6.

[2] Munsky B, Khammash M. The finite state projection algorithm for the solution of the chemical master equation. The Journal of Chemical Physics. 2006;124(4):044104. doi:10.1063/1.2145882.

[3] Carroll L. Sylvie and Bruno Concluded. London: Macmillan and Co.; 1894.

[4] Borges JL. Collected Fictions. Penguin Classics; 1999.

Supplementary Figures

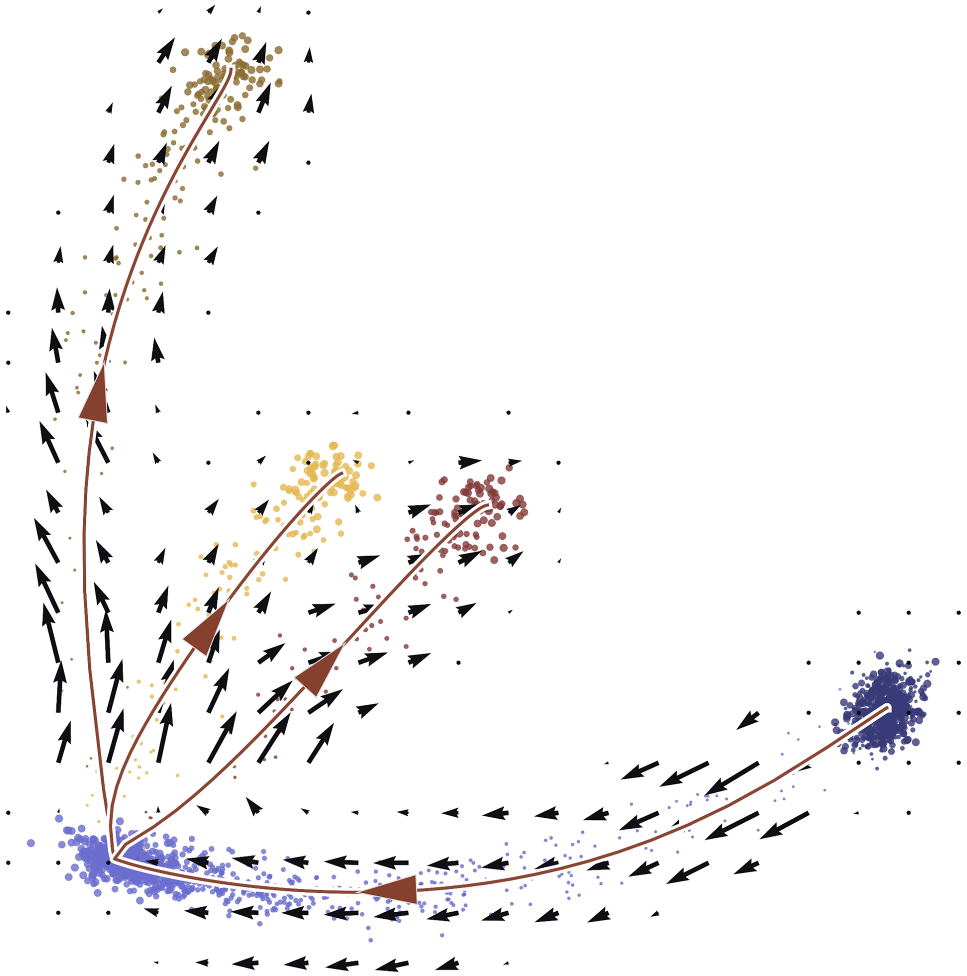

Fig A: RNA velocity, as implemented in *velocyto*, can recapitulate the differentiation trajectories latent in data simulated from a tripotent trajectory. Trajectory directions are displayed to guide the eye (dark blue: cells in the source state; light blue: cells in the intermediate state; yellow, brown, and dark red: cells in one of the terminal states; black arrows: velocity embeddings produced using the standard *velocyto* workflow; lines with arrows: ground truth principal curves).

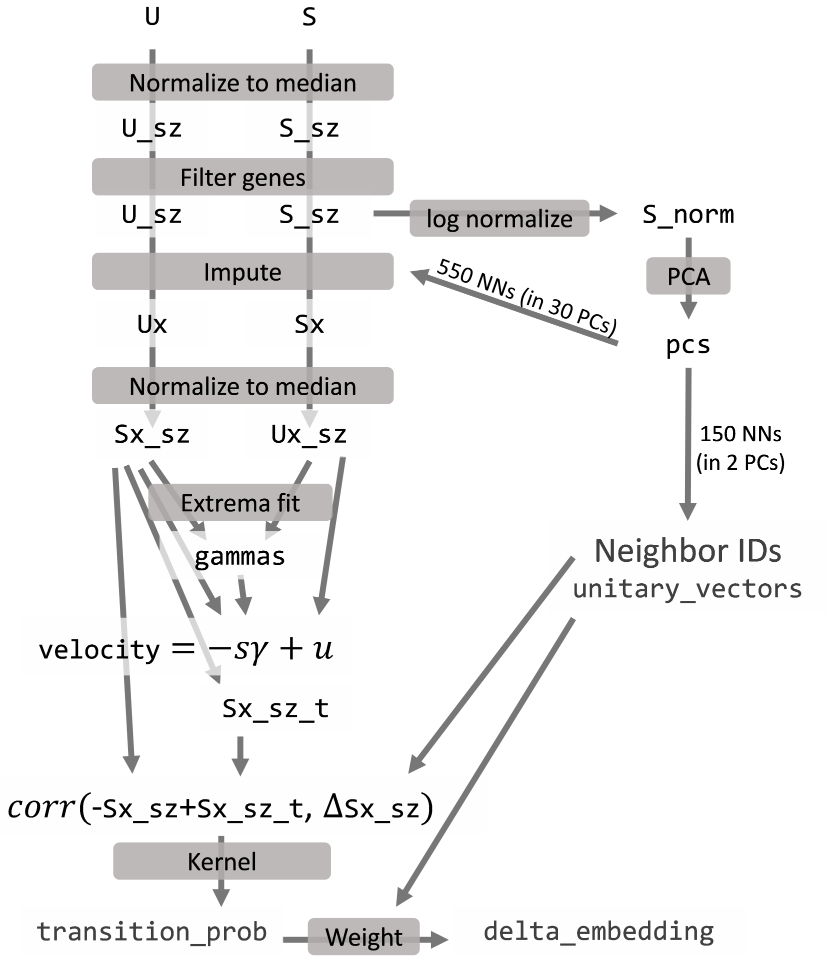

Fig B: A summary of the data manipulations performed in a single run of the *velocyto* workflow.

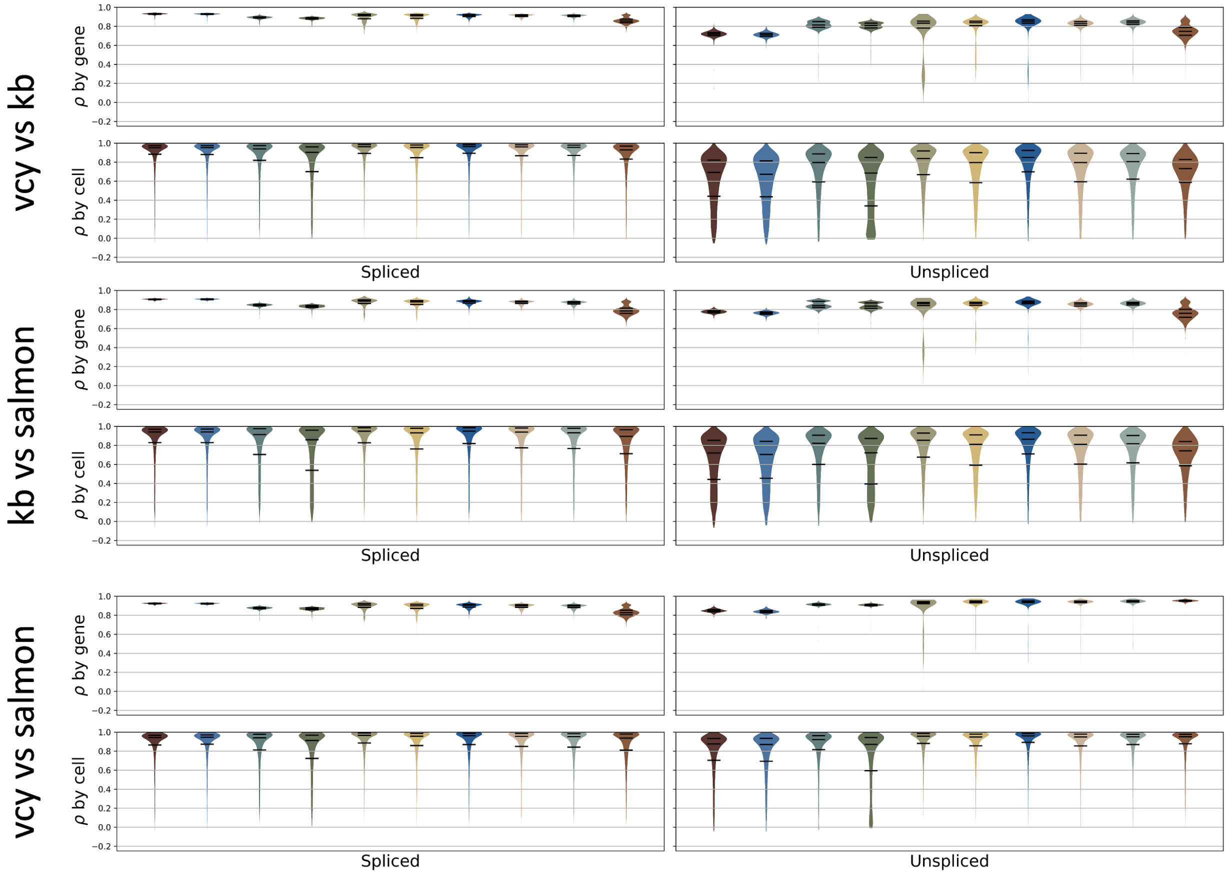

Fig C: Distribution of Spearman correlation coefficients between outputs of *velocyto* (vcy), *kallisto|bustools* (kb), and *salmon*, as obtained from the 10 datasets enumerated in "Data availability." Calculation "by cell" considers each gene in turn and computes correlation over all cells. Calculation "by gene" considers each cell in turn and computes correlation over all genes. Cells that only occur in a single software output and genes observed in fewer than four cells are omitted from analysis (Color: dataset; ρ: Spearman correlation value; violin plot: kernel density estimate of correlation distribution; horizontal lines: 25th percentile, median, and 75th percentile of correlation distribution). The palette used is derived from `dutchmasters` by EdwinTh.

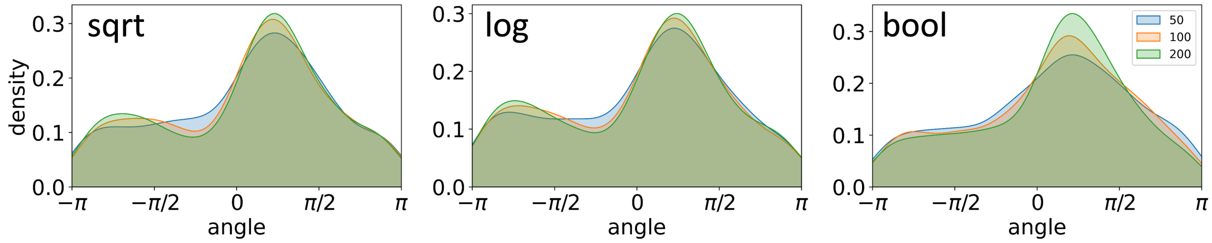

Fig D: Nonlinear transformations and modulation of neighborhood sizes introduce distortions in the arrow directions with respect to the simplest PCA projection (subplots: different transformation procedures applied before kernel density estimation; histograms: distribution of cell-specific angle deviations under different pooling neighborhood sizes k).

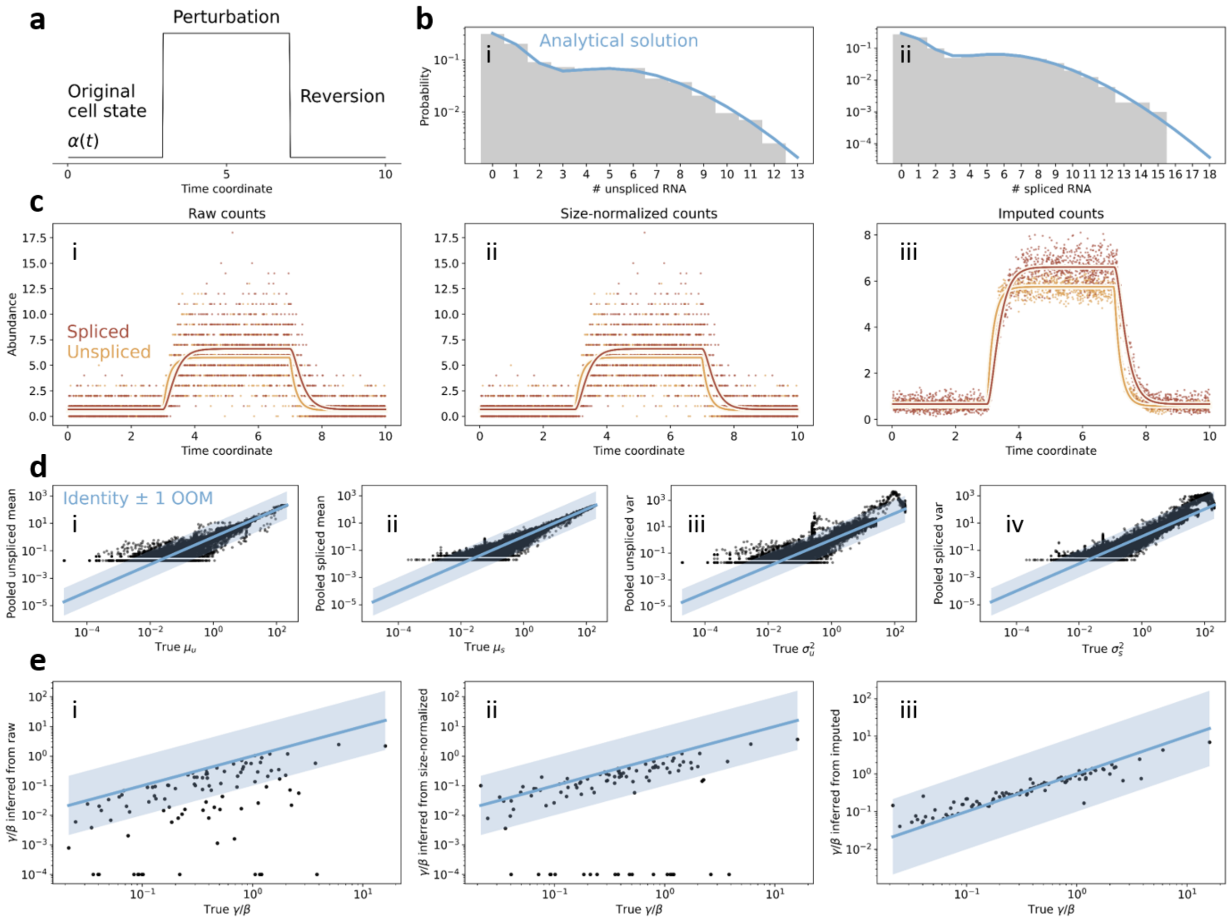

Fig E: The RNA velocity count processing and inference workflow, applied to data generated by stochastic simulation, as in Fig 9 but without normalization with respect to the total number of molecules in the simulated cell. The palette used is derived from `dutchmasters` by EdwinTh.

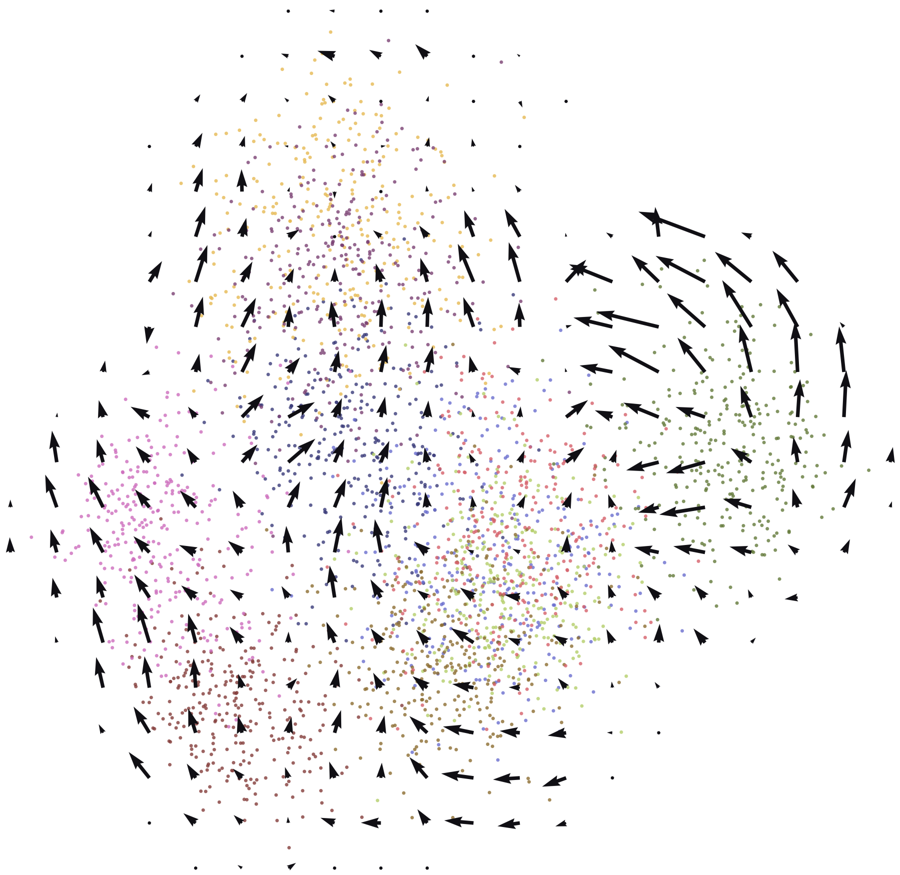

Fig F: Erroneous velocity arrows resulting from a set of ten disjoint cell types with simulated bursty transcription (colors: ground truth cell types; arrows: velocity directions from default nonlinear embedding procedure).

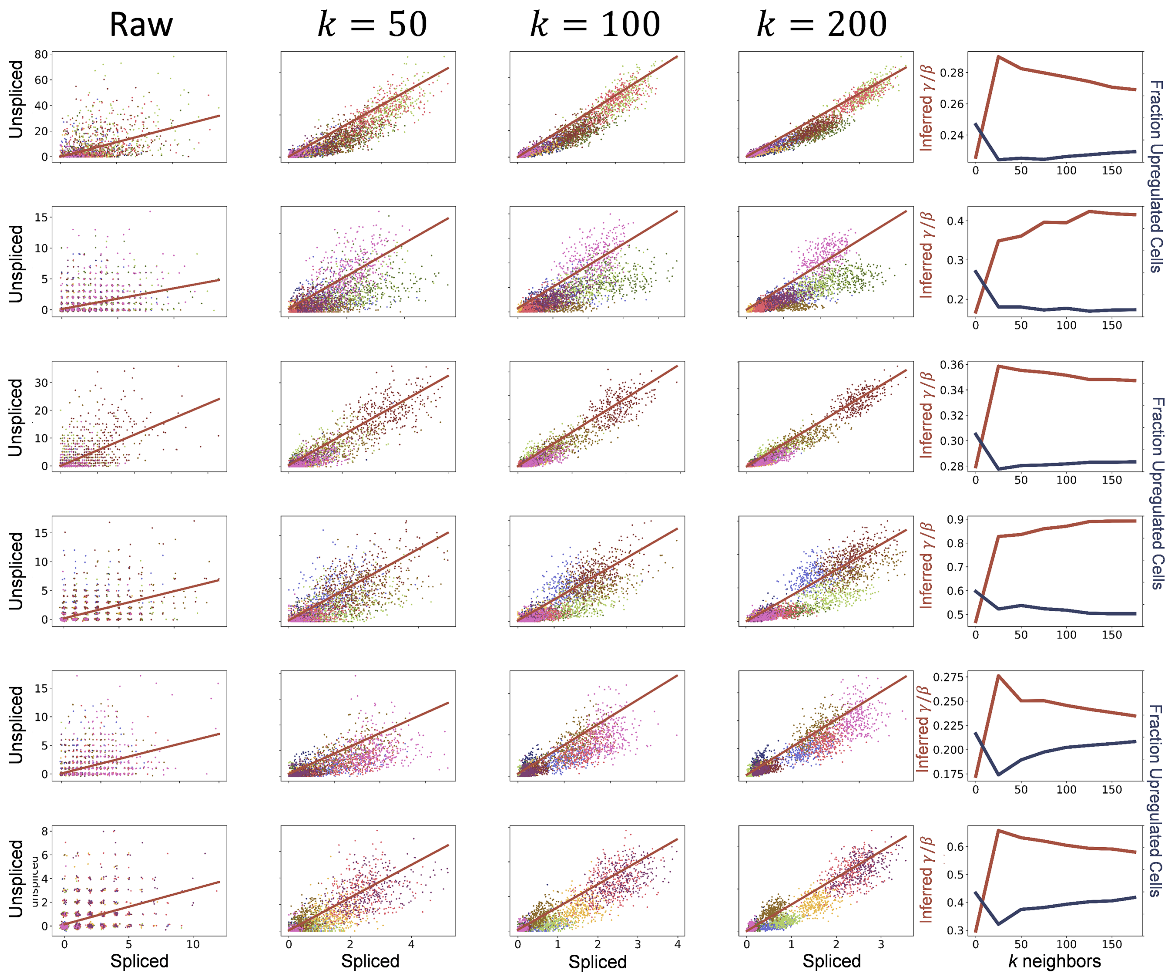

Fig G: Phase plots resulting from applying the standard *velocyto* count processing workflow to data consisting of a set of disjoint cell types with bursty transcriptional dynamics (as in Fig F).
